# Effect of Clamped Member Material and Thickness on Bolt Self-Loosening Under Transverse Loads

**DOI:** 10.3390/ma18020462

**Published:** 2025-01-20

**Authors:** Rashique Iftekhar Rousseau, Abdel-Hakim Bouzid

**Affiliations:** Department of Mechanical Engineering, École de Technologie Supérieure, 1100 Notre-Dame Ouest, Montreal, QC H3C 1K3, Canada; hakim.bouzid@etsmtl.ca

**Keywords:** self-loosening, bolted joint, high-density polyethylene, steel, stiffness

## Abstract

Bolted joints, prevalent in industrial applications for component fastening, are susceptible to self-loosening—a critical issue resulting in a gradual reduction in clamping force. Gaining insight into the underlying mechanisms of self-loosening is crucial. While prior research has largely focused on evaluating component stiffness, limited attention has been given to its impact on the self-loosening behavior of bolted joints under transverse cyclic loading. This study investigates how component stiffness influences self-loosening in bolted joints by varying the material and thickness of clamped members. An experimental setup replicating real-world conditions is devised to simulate loosening caused by cyclic lateral displacement. Tests are conducted using steel and high-density polyethylene (HDPE) clamped members of different grip lengths to explore the relationship between stiffness and self-loosening. Key parameters measured include bolt axial load, transverse force on clamped members, relative displacement, and rotation between the bolt and nut. The findings provide valuable insights into the effects of stiffness across various clamped member materials and grip length combinations, which can enhance the understanding of conditions that promote loosening resistance. Moreover, by highlighting stage-II or rotational loosening, with each test resulting in complete preload loss, the study provides a comparative analysis of the influencing factors. This enables the identification of distinct loosening patterns and supports the development of improved bolted joint designs to reduce loosening.

## 1. Introduction

Despite being widely popular for the use of fastening in most industries due to their ease of installation and removal techniques, bolted joints are likely to be subjected to self-loosening, a major problem that is defined as a gradual decrease in their clamping force. Self-loosening occurs in a clamped joint under various types of loadings, and in particular transverse, vibration, and shock loadings. A tiny imbalance in rotating machines or the influence of dynamic forces in static structures can cause vibrations, by which the contact stress in the engaged threads and in other contact surfaces of the joint components are unbalanced, creating a misalignment between the bolt and nut and causing untightening during sliding of the latter with the adjacent clamped member. Consequently, rotational self-loosening occurs due to this mechanism causing the joint to loosen. Therefore, the joint must have a minimum amount of preload to ensure enough clamping force and structural integrity [1]. However, an excess amount of preload can cause plastic deformation and, hence, notable reduction in joint integrity [2]. There are methods proposed as solutions to prevent the occurrence of self-loosening, such as adding nylock nut, aerotight nut, chemical lock, cheveloc nut, flash washer, etc. [3]. Despite ongoing efforts, no definitive and reliable solution has been developed due to the challenges in controlling the loosening phenomenon and the complex behavior of bolted joints under transverse loading conditions. There are basically two separate stages of loosening. The first one refers to early-stage loosening or non-rotational (stage-I loosening) due to cyclic plastic material deformation happening at the contact surface of the engaged threads and other contact interfaces, which results in the reduction in the clamping force because of stress redistribution as the cycle advances [4]. No relative rotation between the bolt and nut happens in this stage. The subsequent stage of loosening (stage-II loosening), namely rotational or self-loosening, is due to the gradual rotation of the bolt relative to the nut, although the background mechanism is still unclear as various effects can contribute to its occurrence [5]. For example, the loss of initial preload reduces the friction forces in the contact surfaces to a critical level under which the backing-off of the nut becomes obvious. The duration of the external forces also matters as short intervals intensify the frequency of the excitation force. Even a tiny change in the lead angle of threads can enhance quick loosening, which makes coarse-pitch threaded joints more prone to loosening due to the higher internal loosening torque compared to fine-pitch threaded joints. Higher preload in the joint results in greater friction forces in the contact surfaces that increases the corresponding normal forces, thus governing the amount of maximum friction forces. Overall, the factors controlling the backing-off of the nut during the self-loosening stage remain ambiguous. During the transition from non-rotational to rotational loosening, a combination of material plastic deformation and backing-off of the nut may be involved in the loosening process. Jiang et al. [2] experimentally showed the demarcation between these two stages by defining a rotation of 0.5 degree between bolt and nut, although this precise distinction between both stages is difficult to identify in practical applications.

Daadbin and Chow [6] demonstrate the effects of the parameters mentioned above, namely initial preload, applied load duration, contact surface frictions, and thread lead angles on the self-loosening of a joint subjected to impact loadings and their combinations that define the loosening intensity. Junker [7] first discovered by experiment that self-loosening is more obvious when a joint experiences vibration loading perpendicular to the bolt axis, namely transverse loading, compared to axial loading. This brought the idea of transverse slip on the bolt head bearing the contact surface to be the initiation of loosening. On the contrary, the idea of the occurrence of localized thread slip to initiate slight loosening before the bolt head slip came up by Pai and Hess [8,9] with their experimental and three-dimensional finite element (FE) analysis. A study close to the earlier ones mentioned was carried out by Izumi et al. [10], where theoretical and numerical aspects were explored to investigate both tightening and loosening processes of threaded joints. Their investigation included three-dimensional FE modeling and classical theory of solid mechanics. The self-loosening phenomenon in an actual bolted joint and the application of preload considering the interactions of joint components were investigated by Dinger and Friedrich [11], who pointed out the system parameters and the external load profile that affect the localized contact state.

Several experimental and analytical studies have been conducted on self-loosening, investigating different joint parameters and their effects. For example, Nassar and Housari [12] investigated the effect of joint hole clearance and fits between bolt–nut engaged threads on the loosening of a joint subjected to cyclic lateral loading. Zaki et al. [13] performed a couple of experimental studies to demonstrate how the friction coefficients on the bolt bearing and thread contact surfaces, and the conical angle and thread pitch [14], affect the loosening of a preload countersunk bolt when subjected to similar cyclic lateral loading. Later, they developed an analytical model and compared its results with those from a previous experiment conducted using the same test setup [15]. Yang et al. [16] showed analytically and experimentally the effect of increased Young’s modulus and decreased grip length, which increased self-loosening, and both situations needed a greater amount of preload. In another analytical study, they showed the importance of decreasing the hole clearance and increasing thread fits to reduce the transverse amplitude, thus preventing loosening [17].

Recent research also showed the noticeable impact of the materials of a joint component on loosening. Shahin et al. [18] studied the impact of temperature on the performance of a high-density polyethylene (HDPE) flanged joint supported by steel backing rings using FE analysis, and discovered noticeable stress relaxation of the material. Much of the bolt stress relaxation takes place within the first 1200 h, which is also observed earlier, both numerically and experimentally, by Jacobson et al. [19,20]. By experimentally determining the time and temperature dependent mechanical properties of HDPE under isothermal conditions over a one-year service period, they found a significant dependence of flanged joints on temperature, as leakage takes place initially with the temperature increase, and thus recommended the retorquing of bolts within the service period. A thermo-mechanical nonlinear FE analysis including heat transfer and structural modeling was performed by Barsoum et al. [21] to investigate the integrity and performance of a manhole structure of a 78-inch HDPE stub-end, steel ring, and blind flange used with a CNAF (compressed non-asbestos fiber) gasket. They found that factors like stud-bolt pretorque level, internal pressure, and outer temperature strongly influence the manhole integrity and performance.

Although the studies discussed above have explored self-loosening behavior from various perspectives, there remains a notable gap in research analyzing the influence of joint stiffness. In this study, the self-loosening of a bolted joint subjected to transverse loading is observed by analyzing the impact of components stiffness. Investigated parameters are the material and thickness of the clamped members, which affect the stiffness of the individual joint components as well as that of the overall joint, and thus impact the loosening behavior. An experimental setup is designed to replicate the loosening phenomenon due to cyclic transverse loading. The test program includes an investigation of the material effects of the clamped members, namely steel and HDPE, and different grip lengths. The idea is to deepen the understanding of rotational self-loosening by measuring key parameters such as transverse load, displacement amplitude, nut rotation, bolt force, and the number of cycles. This analysis aims to identify distinct loosening patterns influenced by component stiffness, thereby facilitating improvements in bolted joint design to effectively reduce self-loosening.

## 2. Experimental Setup

**Figure 1:**
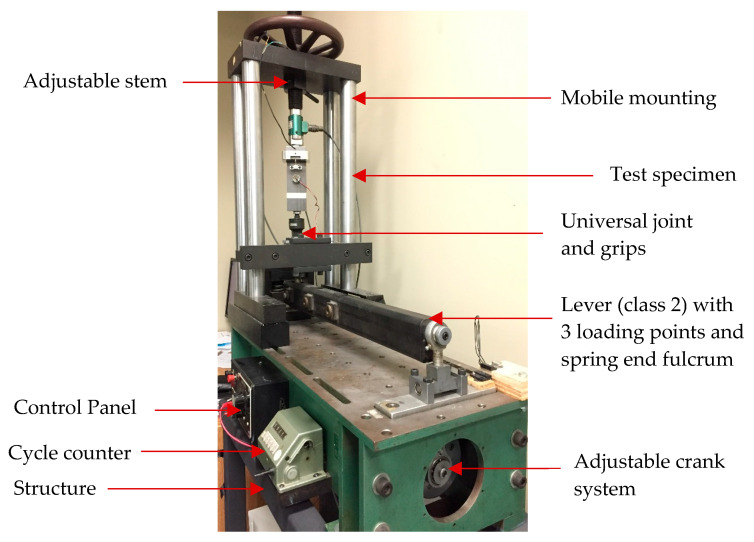
Experimental test rig with M12 × 1.75 hex bolt, nut, and washers.

Figure 1 shows the experimental text rig with its major components developed in the Static and Dynamic Sealing Laboratory which can replicate the self-loosening phenomenon of a bolted joint under cyclic transverse loading. It consists of two clamped members of equal thickness tightened with a bolt, a nut, and two washers.

The instrumented joint system is mounted on an existing direct tension–compression stress fatigue machine (Model DS-6000 HLM, Fatigue Dynamics Inc., MI, USA) that consists of a holding structure, electrical motor, adjustable crank system, basic control panel, grips and universal joint, actuator lever, and an adjustable frame, as shown in the figure. The clamped members with three different thicknesses of 10, 12, and 14 mm are considered in this study, with holes of 13.6 mm diameter to accommodate and fasten an M12 × 1.75 mm hex bolt and nut of grade 8.8. Therefore, the setup can accommodate bolted joints with different grip lengths of 25, 29, and 33 mm including two washers of 2.4 mm thickness each on both sides of the clamped members.

**Figure 2:**
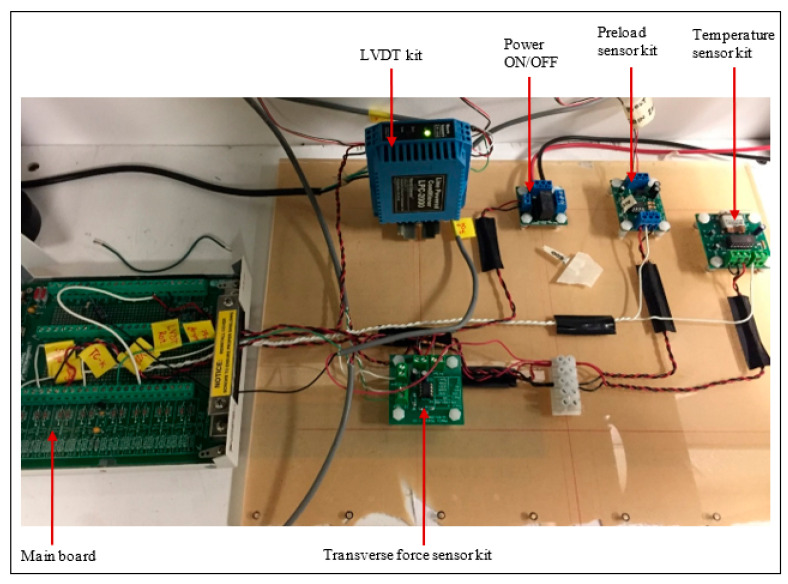
Data acquisition system: main board and electrical components.

To measure the bolt preload, a 1.5 mm diameter cylindrical strain gage (KYOWA KFG-3-120-C20-11, Japan) is installed permanently inside the bolt to avoid adding a load cell that can alter the grip length and joint stiffness. The gage is capable of measuring a wide linear range of axial forces and calibrated with M12 × 1.75 hex bolt below its yield strength. Since the relative rotation between the bolt and the nut is important for the evaluation of stage-II loosening, a rotary variable differential transformer (RVDT CP-2UT, Midori Precisions, Japan) is installed on the nut with three M3 screws and attached to the bolt end through a tongue and groove system. It has an output sensitivity of 2% Vin/10° while measuring ± 45° angle in a linear range. A force transducer (GSE load cell 5410-8k, MI, USA) with a capacity of 8000 lbf is attached within the test setup and perfectly positioned between the clamped members to measure the lateral force. The moving clamped member is connected to an actuator by means of a universal ball joint, which is operated by a crank-drive mechanism. Therefore, the actuator converts the rotational movement of the motor shaft to a reciprocal lateral movement, giving the desired transverse displacement. A spring-loaded flexible plate system installed between the joint specimen and actuator lever helps control the lateral movement. The displacement provided to the actuator through the crank-drive mechanism is not entirely reflected on the clamped member end and a measurement of the relative displacement between the members is required. Therefore, a linear variable differential transformer (Macro CD 375-025 LVDT, Bloomfield Hills, MI, USA) with a nominal range of ±0.63 mm is installed on the moving clamped member close to the bolt to precisely measure the relative displacement. To count the number of loading cycles of the transverse displacement, as well as to stop the test automatically, a magnetic pulse count sensor is installed at the junction of the actuator lever and the connecting crank rod. A data acquisition and control system unit from National Instrument is connected to the test bench by means of a computer and programmed with the LabView 2019 software to directly monitor and record the data (Figure 2).

**Figure 3:**
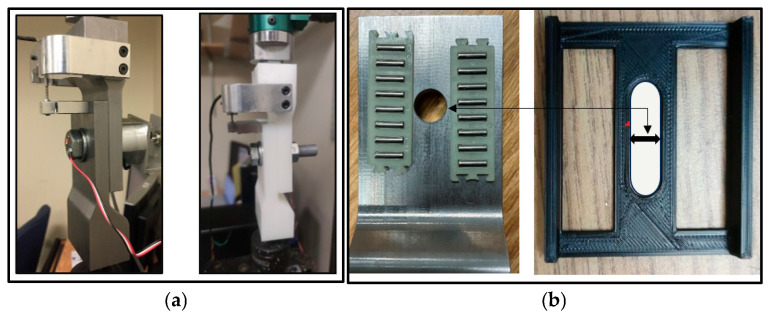
(**a**) Steel and HDPE clamped members; (**b**) Roller bearings (INA-HYDREL FE series) on contact face of steel clamped members and 3D printed hard plastic housing.

In this study, the test samples for the clamped members are made from two different materials. Steel and high-density polyethylene (HDPE) materials were selected to investigate the effect of materials on the behavior of self-loosening. For a particular test case with higher preloads for steel clamped members of different thicknesses, two single-row flat roller bearings (INA-HYDREL Flat Cage Assemblies FE series, BC, CA) are placed between the clamped members to reduce the initial friction in the joint at the beginning of loading cycles. Figure 3a illustrates a 3D printed roller cage housing made of hard plastic, which is used to properly position and align both roller bearings. The dimensions of the rectangular slots and the hole are carefully maintained during the 3D printing so that the bolt is inserted easily and both roller bearing fits perfectly into the slots without titling when the components are clamped and tightened together (see Figure 3b).

The self-loosening test is conducted by providing lateral displacement to the moving clamped member since the test rig is controlled on displacement. The joint is preloaded to achieve the desired clamping force before starting the cyclic transverse movement using the electrical motor, generally with a low frequency of around 1 Hz. The tests are conducted until a significant drop of preload is observed.

The mechanical properties of the grade 8.8 steel bolt and clamped members made of AISI 1045 cold drawn steel and HDPE are given in Table 1. Table 2 provides some experimental test data conducted on both steel and HDPE bolted joints with different preloads and amplitudes of lateral displacement. The applied load is limited by the strength of the clamped member material in the case of HDPE and the maximum transverse load allowed by the test setup. The amplitude is increased gradually until self-loosening takes place.

## 3. Results and Discussion

### 3.1. Effect of Joint Stiffness

The stiffness of a joint depends not only on the thickness of the clamped members but also on the material they are made of. The thinner the clamped members, the stiffer they become, which can be appreciated by the formulae given in [22,23]. Both parameters are investigated hereafter.

#### 3.1.1. Effect of the Thickness of the Clamped Members

**Figure 4:**
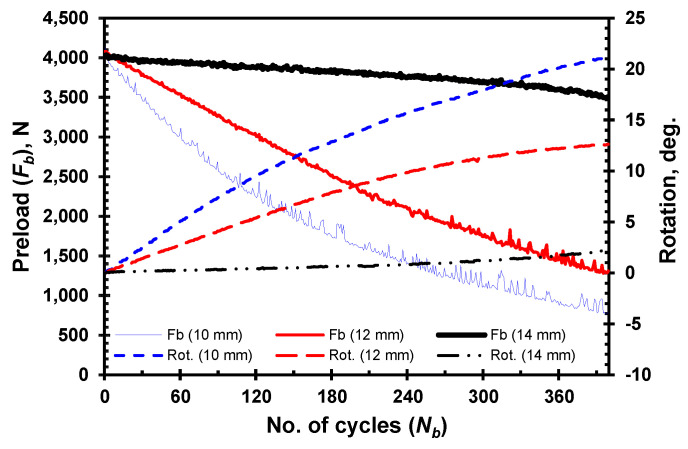
Loosening by preload drop and bolt–nut relative rotation for HDPE clamped members of different thickness.

A series of experiments are conducted to examine how the thickness of the clamped members influences the self-loosening behavior of bolted joints. Figure 4 illustrates the loosening behavior by displaying the change in preload drop and relative rotation between the bolt and nut for the HDPE clamped members, with thicknesses of 10, 12, and 14 mm as loading cycles progress. In this case, the joint is tightened with a lower initial preload of 4 kN due to the use of HDPE clamped members. Self-loosening is more prominent in joints with thinner clamped members, showing a quicker preload drop as the number of load cycles increases. A similar pattern of stage-II loosening is evident, with an increased rate of rotation between the bolt and nut in joints with thinner clamped members. Both plots clearly show that stiffer joints, specifically those with thinner clamped members, tend to loosen more quickly. Thus, the thickness of the clamped member plays a crucial role in the loosening behavior of the joint, directly affecting its stability. The stiffness of bolted joints is a key design factor that must be considered to enhance their performance, reliability, and longevity.

**Figure 5:**
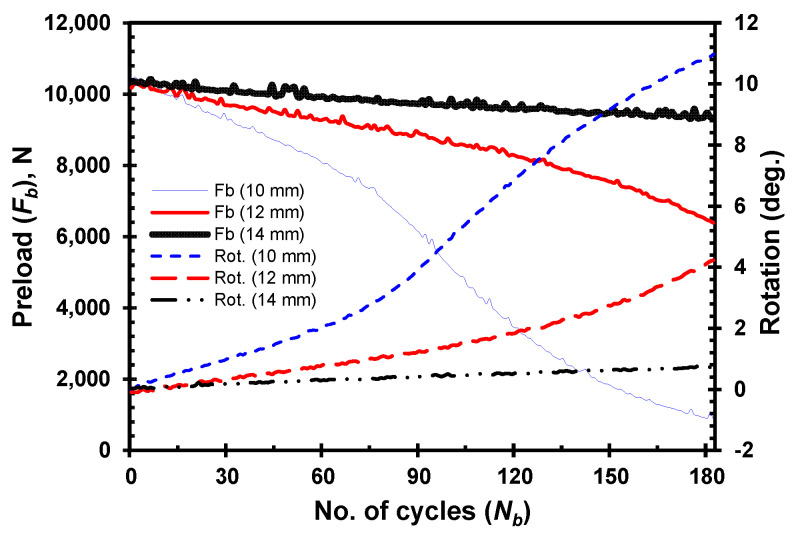
Loosening by preload drop and bolt–nut relative rotation for steel clamped members of different thickness.

Figure 5 depicts the loosening behavior by showing the loss of preload when the joint is tightened with a higher preload of 10 kN for the same thickness range of the steel clamped members, as in the case of HDPE. As previously observed, joints with thicker clamped members retain preload more effectively than those with thinner members. The relative rotation between the bolt and nut is greater in joints with thinner clamped members, due to their higher stiffness.

#### 3.1.2. Effect of the Material of the Clamped Members

**Figure 6:**
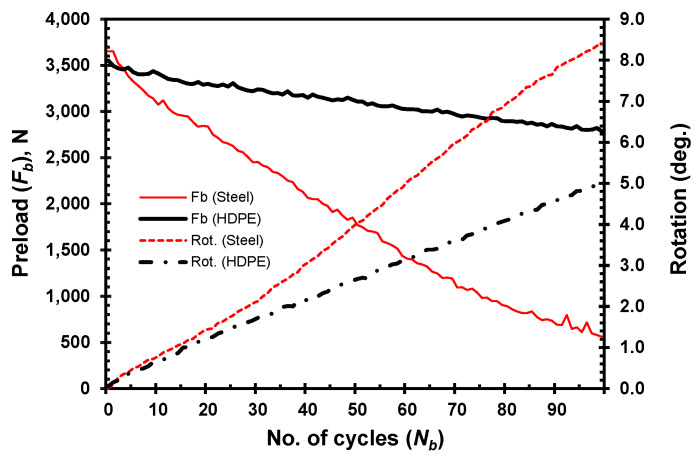
Preload drop and bolt–nut relative rotation with 10 mm HDPE and steel clamped members.

Self-loosening of bolted joints is highly dependent on the materials selected for the clamped members. A few identical sets of steel and HDPE clamped members are chosen for this analysis. To maintain similar test conditions, the joints with steel clamped members do not include flat roller bearing as in earlier tests. Therefore, a lower preload of 3.5 kN is chosen to tighten both joints for the tests. Figure 6 shows the results of two tests conducted on bolted joints with clamped members of 10 mm thickness for both materials. Both bolted joints underwent 100 transverse displacement cycles with an amplitude of 0.2 mm. As shown in the figure, the joint with steel members has less capability of retaining preload. It loses preload rapidly, since it exhibits higher loosening rate with the number of cycles. However, bolted joints with HDPE clamped members exhibit greater resistance to self-loosening by maintaining the initial tension more effectively throughout the entire test. The relative rotation between the bolt and the nut is higher for steel joints as compared to HDPE joints, allowing a faster occurrence of rotational loosening comparatively. The results of Figure 6 clearly indicate that the joints with stiffer clamped members are more likely to undergo rapid loosening. This provides insight into material selection when designing bolted joints for practical applications.

**Figure 7:**
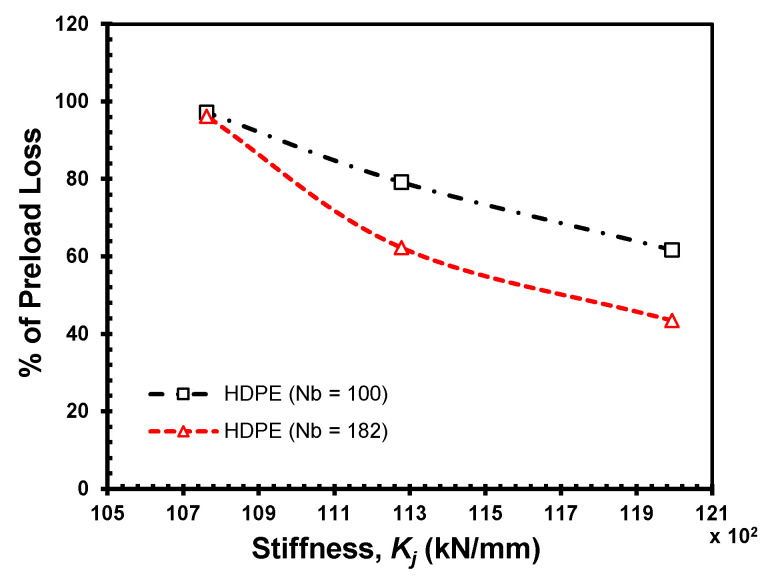
Self-loosening by preload loss at different stiffness points for HDPE joints.

**Figure 8:**
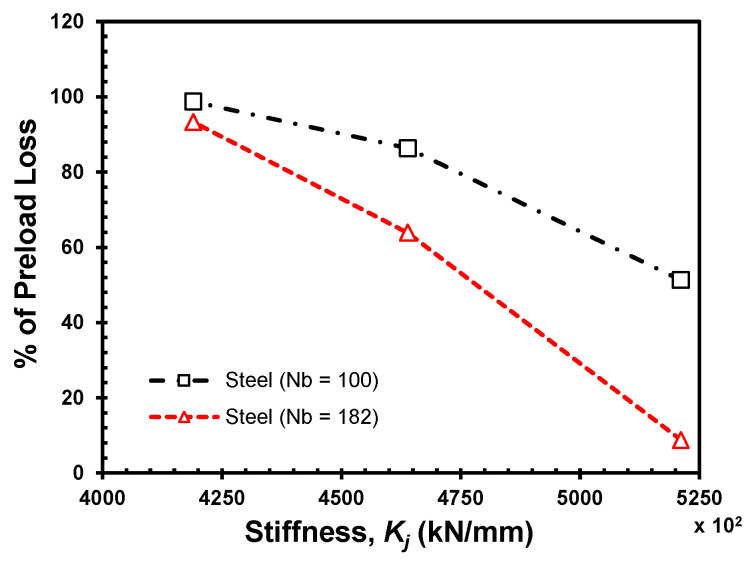
Self-loosening by preload loss at different stiffness points for steel joints.

To illustrate the combined effect of the thickness and material, the results are grouped together to present the load drop as a function of the stiffness in Figure 7 and Figure 8. Figure 7 shows self-loosening by the loss of initial preload of 4 kN at different stiffness points for joints with HDPE clamped members at 100th and 182nd loading cycles. The amplitudes of lateral displacement at the two selected cycles remain constant for each grip length. The consistent displacement levels indicate that HDPE joints have a stronger resistance to self-loosening.

For steel joints with an initial preload of 10 kN, loss of preload is bigger with increased stiffness, i.e., decreased grip length at selected loading cycles, as shown in Figure 8.

Overall, both materials maintain better bolt preload at lower stiffness values, while loosening becomes more pronounced as the stiffness increases. The self-loosening behavior of joints with HDPE and steel clamped members is challenging to compare directly due to differing lateral movement amplitudes at the beginning and throughout the loading cycles. However, joints with steel clamped members are noticeably more rigid and experience preload loss more frequently than HDPE joints. Although preload drop occurs in both joints, steel joints exhibit a slightly higher rate of preload loss resulting in self-loosening, even with a lateral displacement amplitude that is ten times lower than that of HDPE, as shown in Table 2.

### 3.2. Effect of the Cycles on Transverse Force

**Figure 9:**
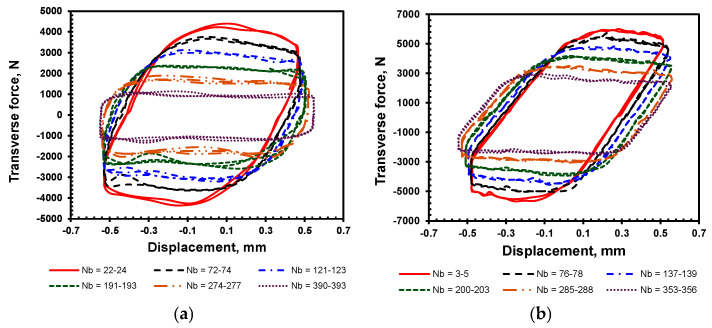
Effect of transverse force on (**a**) 10 mm and (**b**) 12 mm HDPE joint at different cycle range.

Figure 9a illustrates the behavior over several loading cycles, highlighting the influence of transverse force on the joint as it undergoes self-loosening until the complete loss of preload. A similar pattern is observed across all loop shape curves within the selected ranges of loading cycles, each characterized by two oblique lines and two almost horizontal lines. The oblique lines are an increase in the lateral force from negative to positive with the displacement and represent the bending of the bolt due to the transverse force of the clamped member, with no sliding between the contact surfaces of the bolt/nut and the clamped members. The horizontal part of the curve begins when the transverse force reaches a threshold equating the friction force during the loading cycle, allowing sliding at the contact surfaces between the bolt head or nut bearing surfaces and clamped members, thereby causing relative rotation between the bolt and nut.

Figure 9b shows a similar behavior trend of the transverse force with a bolted joint having 12 mm HDPE clamped members. The transverse force, which depends on the friction at all interacting contact surfaces, diminishes over cycles because the bolt force also decreases with each cycle. Nevertheless, the transverse force is shown greater for the 12 mm clamped members when compared to that for 10 mm because the load is relatively higher at the given cycles shown in Figure 9, for the same initial preload of 4 kN, coefficient of friction (*µ* = 0.22), and a lateral displacement of 0.5 mm.

### 3.3. Effect of the Amplitude of Lateral Displacement

**Figure 10:**
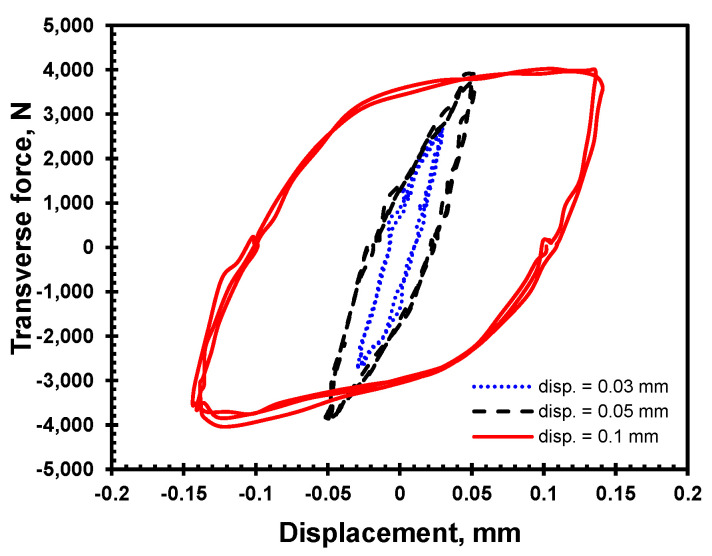
Effect of amplitude of lateral displacement on joints with steel clamped members.

Figure 10 illustrates the effect of varying the amplitude of the lateral displacement on a bolted joint with steel clamped members and roller bearings. The initial preload is set at 10 kN. As in the previous section, the two distinct oblique lines are still present during the loading cycles. The slope of these lines represents bolt bending stiffness. However, the slope of the second part of the curve is small and is related to the sliding between the engaged thread contact surfaces, causing the self-loosening by bolt-to-nut relative rotation.

## 4. Conclusions

An experimental study is performed to show the influence of joint stiffness on the self-loosening of a bolted joint subjected to transverse loading. Effects of different parameters on loosening, such as the thickness and material of the clamped members, are studied. The impact of the amplitude of lateral displacement and the transverse force are shown to have a big influence. A greater resistance to self-loosening is observed when the joints have thicker clamped members as they have lower stiffness. The rotation of the nut relative to the bolt increases with the number of load cycles and amplitude. Also, HPDE clamped members having lower stiffness retain better initial preload in the joint compared to steel counterparts. Two distinct self-loosening phases are observed in clamped joints during certain loading cycles: first, initial bolt bending with sliding between clamped members but not yet at the bolt and nut contact surfaces, until a critical transverse displacement is reached; second, sliding begins at the thread contact surfaces. Self-loosening in HDPE joints of different thicknesses is also observed by analyzing the transverse force required for the same lateral movement.

Incorporating additional industry-standard materials relevant to the application could expand the understanding of self-loosening and enhance the knowledge of input conditions necessary for improving loosening resistance. Furthermore, examining the effect of creep on the relaxation of clamping force in high-temperature applications could introduce a new dimension to this research.

## Figures and Tables

**Table 1:** Mechanical properties of bolt (grade 8.8) and clamped members of steel (AISI 1045 cold drawn) and HDPE.

	Bolt Grade 8.8	Clamped Member	
		Steel (AISI 1045 Cold Drawn)	HDPE
Ultimate tensile strength (MPa)	800	585	30
Yield strength (MPa)	640	450	21.9
Young’s modulus (GPa)	206.8	206	0.995
Poisson’s ratio	0.3	0.3	0.45
Coefficient of Friction as reference	0.12	0.738	0.2

**Table 2:** Experimental test data for HDPE and steel joints with different preloads and initial amplitudes of lateral displacement.

Material	Thickness (mm)	Preload (kN)	Amplitude (mm)
Steel	10	10	0.05
	12	10	0.05
	14	10	0.04
HDPE	10	4	0.5
	12	4	0.49
	14	4	0.38

## Data Availability

The data presented in this study are available on request from the corresponding author due to privacy.
